# An Abnormal Presentation of Age-Related Macular Degeneration in a Young, Healthy Adult

**DOI:** 10.7759/cureus.90672

**Published:** 2025-08-21

**Authors:** Millena Rodrigues De Moura, Tianna Sasher, Travis Smith

**Affiliations:** 1 Family Medicine, Lake Erie College of Osteopathic Medicine, Bradenton, USA; 2 Ophthalmology, Lake Erie College of Osteopathic Medicine, Bradenton, USA; 3 Clinical Curriculum Integration and Assessment, Lake Erie College of Osteopathic Medicine, Bradenton, USA

**Keywords:** age-related macular degeneration, eye screening, health screening, macular degeneration, ophthalmology, primary care

## Abstract

Age-related macular degeneration (AMD) is a relatively uncommon diagnosis in patients under the age of 45. It typically manifests as a gradual or sudden loss of central vision, producing blurred images or wavy visual distortions. These symptoms can interfere not only with daily activities but also with more detailed visual tasks, significantly impacting quality of life. This case report illustrates the rare presentation of intermediate AMD in a 43-year-old male with no significant past medical history. The case also highlights the importance of maintaining routine health examinations, including annual primary care visits and baseline ophthalmologic examinations. Regular health screenings are vital for the early detection of potential diseases and are a crucial part of preventive medicine.

## Introduction

Age-related macular degeneration (AMD) is a progressive, chronic condition that involves loss of central vision, resulting in permanent visual impairment [1,2]. It is recognized as the third leading cause of blindness worldwide [1]. Although AMD does not cause complete vision loss, it continues to worsen central vision and makes it difficult to drive, work, and visualize faces and objects. There are two types of AMD: dry (atrophic) and wet (neovascular) [1,2]. Dry AMD is the most common type and occurs in three stages: early, intermediate, and late. The early stage involves medium-sized drusen deposits without vision loss [3]. In the intermediate stage, patients present with medium-sized drusen and pigmentary abnormalities [2]. The late stage, also known as geographic atrophy, involves round areas of hypopigmentation, depigmentation, or absence of the retinal pigment epithelium [4-6]. Wet AMD occurs when abnormal blood vessels proliferate under the retina, leading to bleeding and fluid accumulation in the macula [7]. It is the less common type of AMD that leads to vision loss at a faster pace. Multiple risk factors are associated with AMD, with age being the most significant demographic factor [2]. Patients at risk include those who are over the age of 60, have a family history of AMD, smoke or have previously smoked cigarettes, are overweight or obese, and have high blood pressure [1,2,8]. According to the National Vision and Eye Health Surveillance System (VEHSS), the percentage of US residents with AMD at any stage between the ages of 40 and 44 was 2.02% in 2019 [9]. Despite this small percentage, the American Academy of Ophthalmology (AAO) recommends a baseline eye disease screening at the age of 40 for patients with no changes to their vision or risk factors [10].

## Case presentation

**Figure 1:**
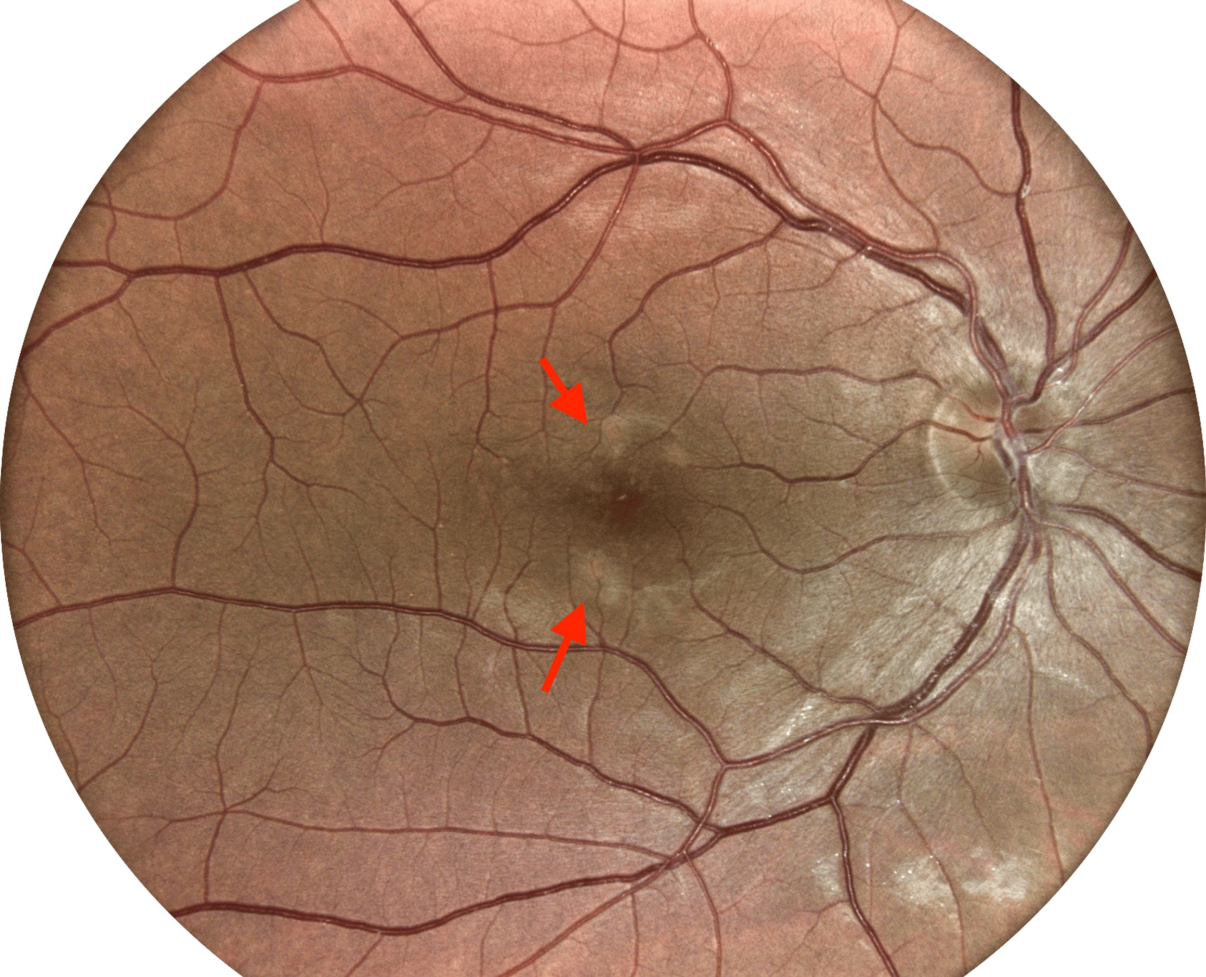
Digital Retinal Imaging of the Right Eye From the Initial Optometry Visit Digital retinal image from the initial optometry visit, with arrows indicating mild-to-moderate hypopigmented macular changes in the right eye.

**Figure 2:**
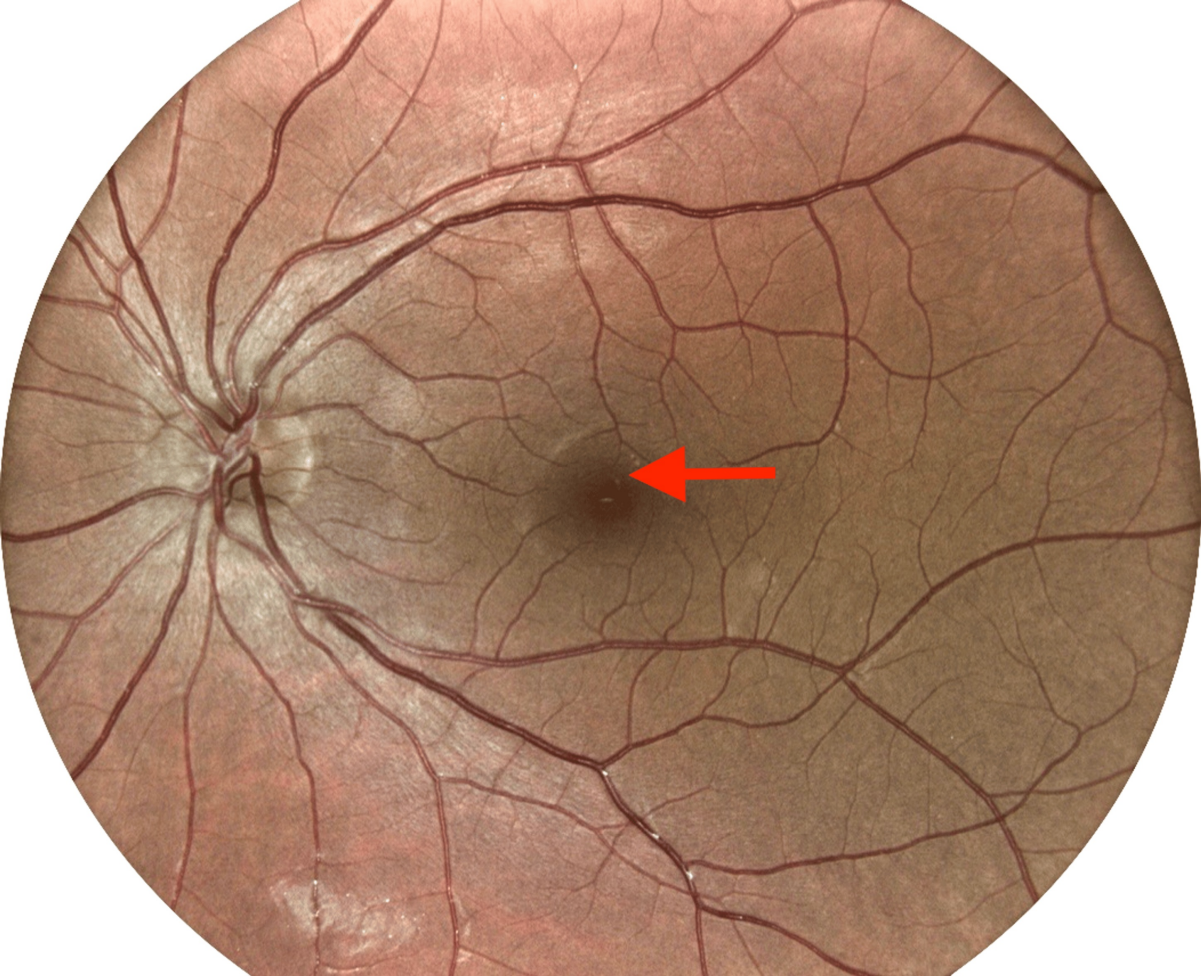
Digital Retinal Imaging of the Left Eye From the Initial Optometry Visit Digital retinal image from the initial optometry visit, with arrows indicating trace hypopigmented macular changes in the left eye.

A 43-year-old male with no past medical history presented to his primary care physician (PCP) with macular changes noted on a routine exam by his optometrist. He stated that, at the age of 37, he noticed that traffic signs were blurry from a distance, and his wife encouraged him to see an optometrist to get prescription glasses. At the time, digital retinal imaging revealed no abnormalities, and he was given a prescription to correct his myopia. He expressed that he only decided to go to the optometrist again because his wife wanted to ensure that his vision remained stable, as he only used his glasses on rare occasions. A new repeat digital retinal imaging performed six years later at his optometry visit revealed significant drusen in his right eye (Figure 1) and slight macular changes in his left eye (Figure 2). He admitted to having trouble visualizing objects at a distance but did not feel the need to wear his glasses often. He denied floaters, flashes, pain, photophobia, or metamorphopsia (wavy distortions of vision). The patient also reports no history of ocular trauma. The optometrist advised him to see his PCP and an ophthalmologist.

Upon examination by his PCP, the patient was awake, alert, and oriented to person, place, and time. His blood pressure was 114/72 mm Hg, heart rate was 68 beats per minute, respiratory rate was 16 breaths per minute, oxygen saturation was 98%, weight was 168 lbs, height was 69 inches, and body mass index was 26.3. Physical examination revealed a 43-year-old male without acute distress. Pupils were equal, round, and reactive to light and accommodation; extraocular movements (EOM) were intact; and there was no nystagmus or periorbital redness or swelling. His heart, lung, and abdominal examinations were negative for any additional findings.

Labs were ordered and significant for elevated low-density lipoprotein (LDL) (122), decreased high-density lipoprotein (HDL) (39), and a prediabetic glycated hemoglobin test (HbA1c) (5.8). Total cholesterol and triglycerides were within normal limits (178 mg/dL and 132 mg/dL, respectively). Both the CBC and the comprehensive metabolic panel (CMP) were unremarkable. The patient had no known medical conditions. His family history was significant for diabetes and hyperlipidemia in his father. He stated that he had never smoked and did not drink alcohol. He was not on any medications and had no known allergies. The patient also reported no family history of AMD or other ocular pathologies. He had no past surgical history and had never been hospitalized. The patient stated he overall felt healthy, maintained a good diet during the week, indulged in sweets on weekends and special occasions, and exercised five times per week.

**Figure 3:**
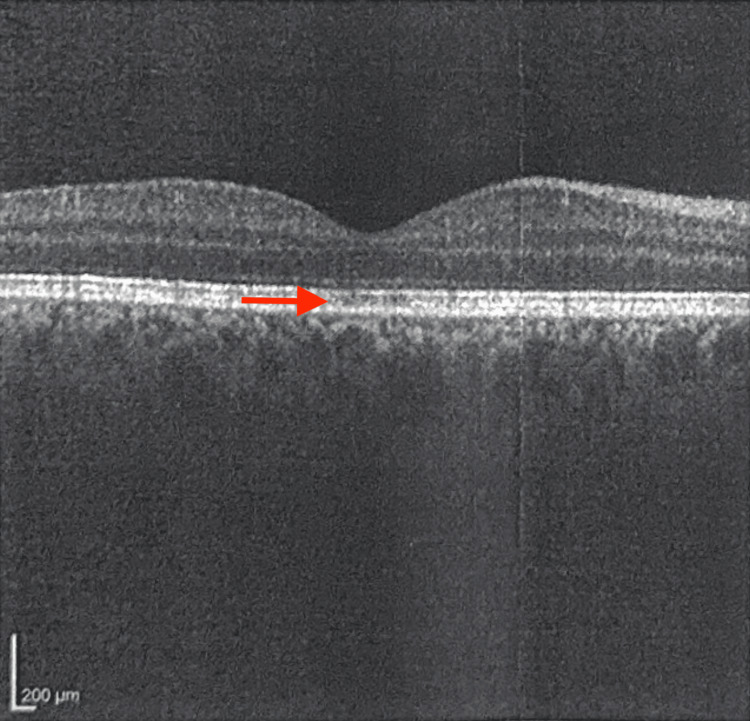
Macular OCT of the Right Eye From the Initial Ophthalmology Visit OCT = optical coherence tomography OCT of the right macula obtained during the patient’s initial ophthalmology visit. The arrow indicates subtle atrophy of the RPE within the fovea.

**Figure 4:**
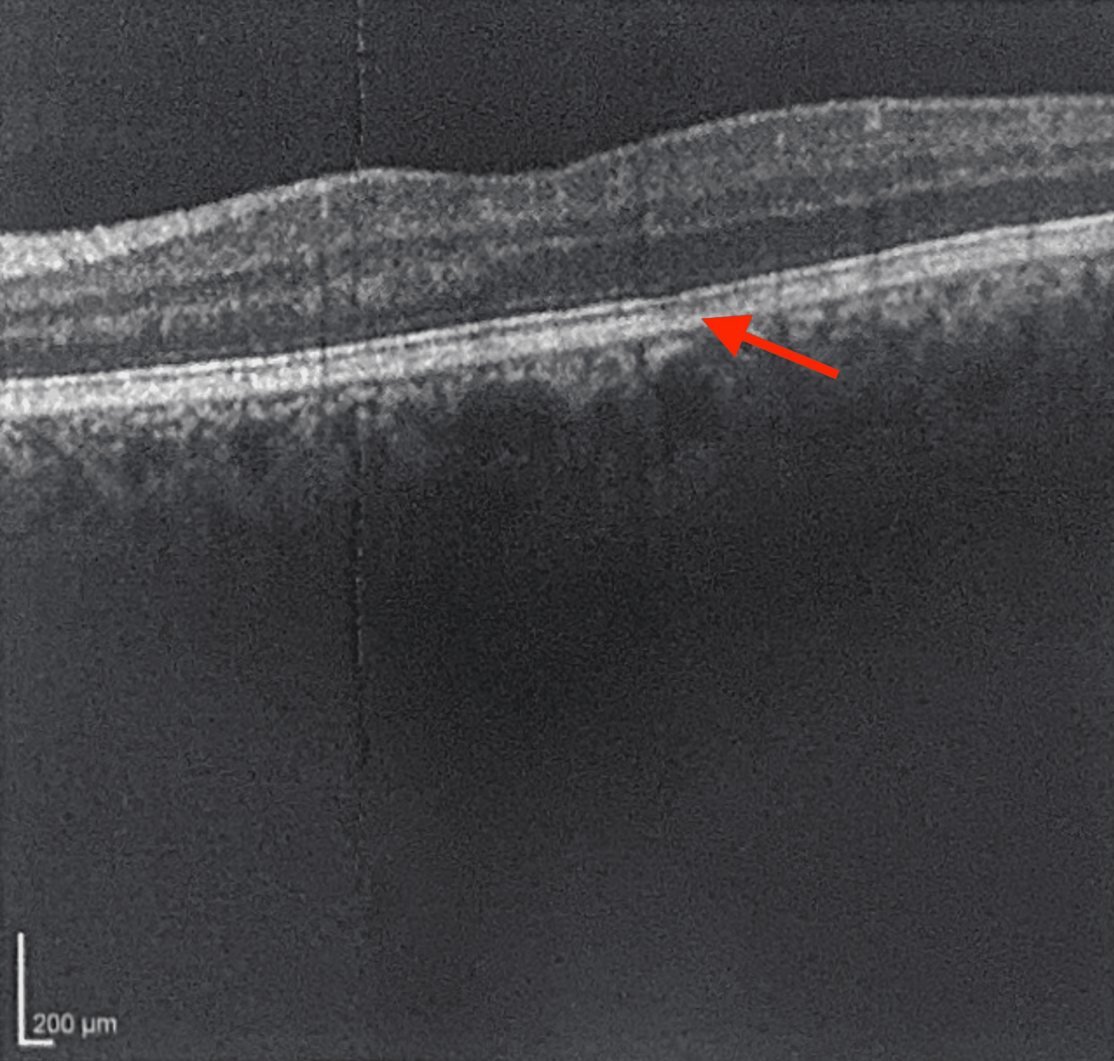
Macular OCT of the Left Eye From the Initial Ophthalmology Visit OCT = optical coherence tomography OCT of the left macula obtained during the patient’s initial ophthalmology visit. The arrow indicates subtle atrophy of the RPE within the fovea and macula.

**Figure 5:**
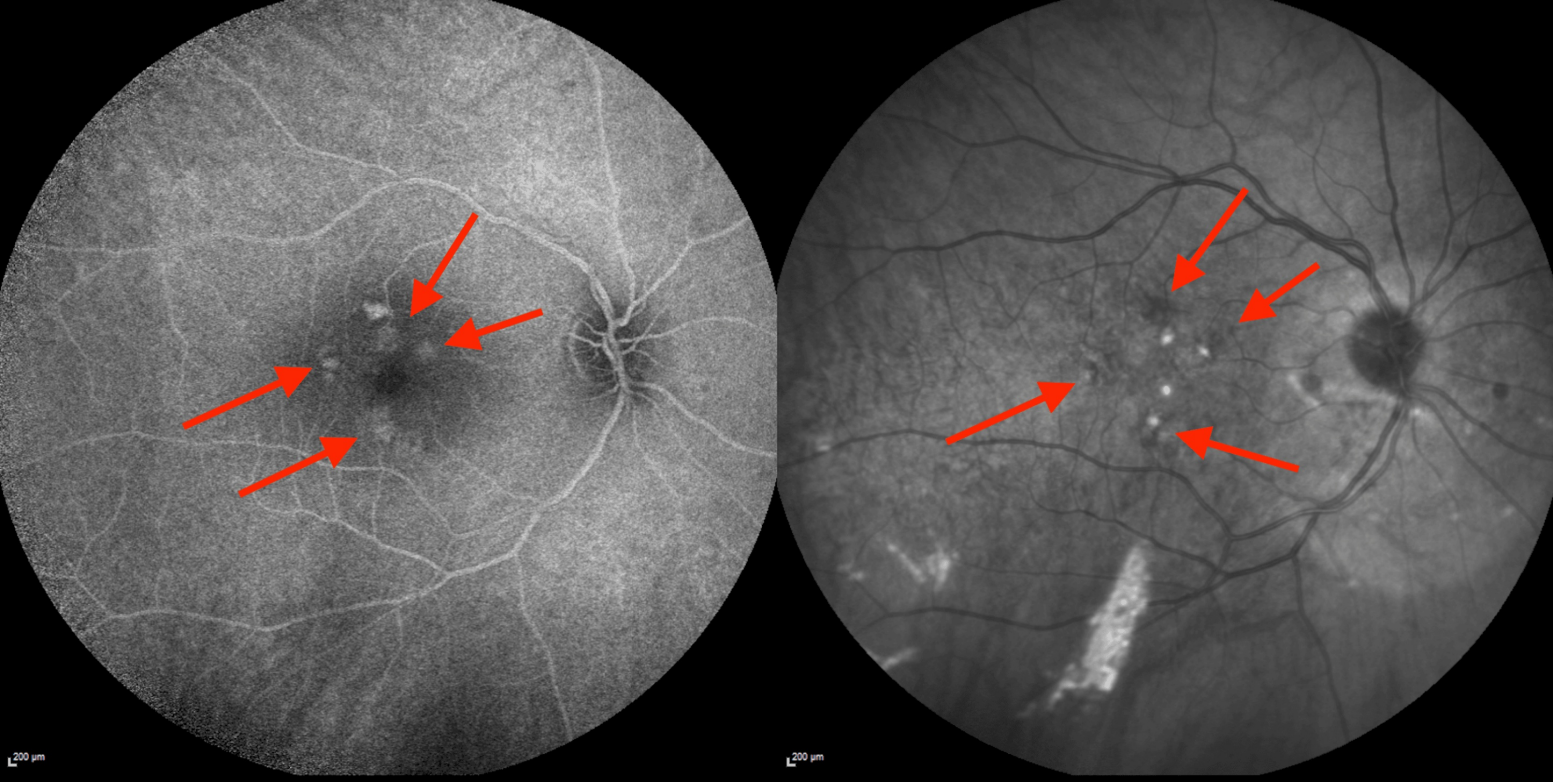
Fluorescein Angiography of the Right Eye From the Initial Ophthalmology Visit Fluorescein angiography of the right eye obtained during the initial ophthalmology visit. Arrows indicate mild-to-moderate perifoveal hyperreflective and hyporeflective lesions.

**Figure 6:**
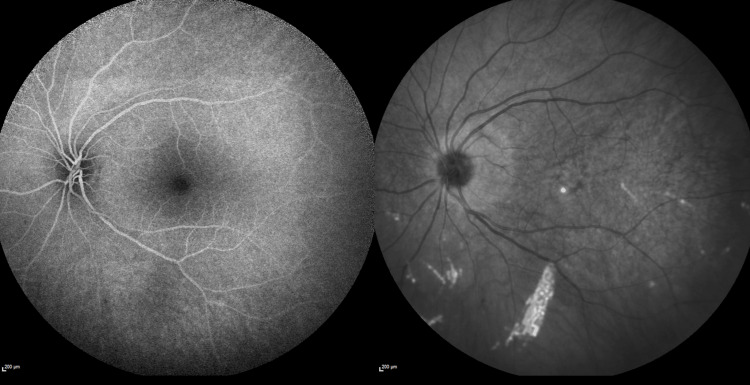
Fluorescein Angiography of the Left Eye From the Initial Ophthalmology Visit Left eye fluorescein angiography from the initial ophthalmology visit demonstrates a largely normal fundus and macula, with no significant lesions to highlight.

The patient was referred to an ophthalmologist, who performed a variety of diagnostic exams, including a baseline eye exam (Table 1), optical coherence tomography (OCT) of the macula (Figures 3-4), and fluorescein angiography (Figures 5-6). Visual acuity in the right eye (OD) was 20/50-2 without correction, and with pinhole (PH) it was 20/20-1. Left eye (OS) visual acuity was 20/40 without correction, and PH was 20/20-1. The intraocular pressures, external exam, and anterior segment exams were all within normal limits. On fundus exam, cup-to-disc ratios were 0.3 in both eyes (OU). The macula in the right eye demonstrated age-related macular degeneration and retinal pigment epithelial (RPE) detachment. The left macula also showed age-related macular degeneration. Vitreous floaters were noted in both eyes, and the periphery was flat with no holes or tears OU.

**Table 1:** Comprehensive Eye Examination Results for Both Eyes at the Initial Ophthalmology Visit OD = oculus dexter (right eye); OS = oculus sinister (left eye); SC = spectacle correction; PH = pinhole; IOP = intraocular pressure; EOM = extraocular movements; CVF = confrontational visual field; VF = visual field; PERRLA = pupils equal, round, reactive to light and accommodation; APD = afferent pupillary defect; CONJ = conjunctiva; NVI = neovascularization of the iris; C/D = cup-to-disc ratio; PVD = posterior vitreous detachment. This exam shows normal anterior and posterior segment findings bilaterally, with AMD noted in both eyes and no signs of acute pathology.

Variables	Right Eye (OD)	Left Eye (OS)
Visual Acuity	SC: 20/50-2	SC: 20/40
	PH: 20/20-1	PH: 20/20-1
IOP	14	12
External Exam	EOM: Full in all gazes	EOM: Full in all gazes
	CVF: Full VF	CVF: Full VF
	Pupils: PERRLA, no APD	Pupils: PERRLA, no APD
	Adnexa: Normal	Adnexa: Normal
	Orbit: Normal	Orbit: Normal
Anterior Segment Exam	Conj/Sclera: Clear and transparent	Conj/Sclera: Clear and transparent
	Cornea: Clear	Cornea: Clear
	Anterior Chamber: Deep and quiet	Anterior Chamber: Deep and quiet
	Iris: Round, flat, no NVI	Iris: Round, flat, no NVI
	Lens: Clear	Lens: Clear
Fundus Exam	C/D: 0.3	C/D: 0.3
	Disc: Sharp and pink	Disc: Sharp and pink
	Macula: Age-related macular degeneration, retinal pigment epithelium detachment	Macula: Age-related macular degeneration
	Periphery: Flat, no holes or tears	Periphery: Flat, no holes or tears
	Vessels: Normal caliber and tortuosity	Vessels: Normal caliber and tortuosity
	Vitreous: Floaters, no PVD, no cells	Vitreous: Floaters, no PVD, no cells

He was diagnosed with nonexudative AMD bilaterally, with an intermediate dry stage in the right eye and an early dry stage in the left eye. He was also diagnosed with serous detachment of the retinal pigment epithelium in the right eye and vitreous degeneration bilaterally.

**Figure 7:**
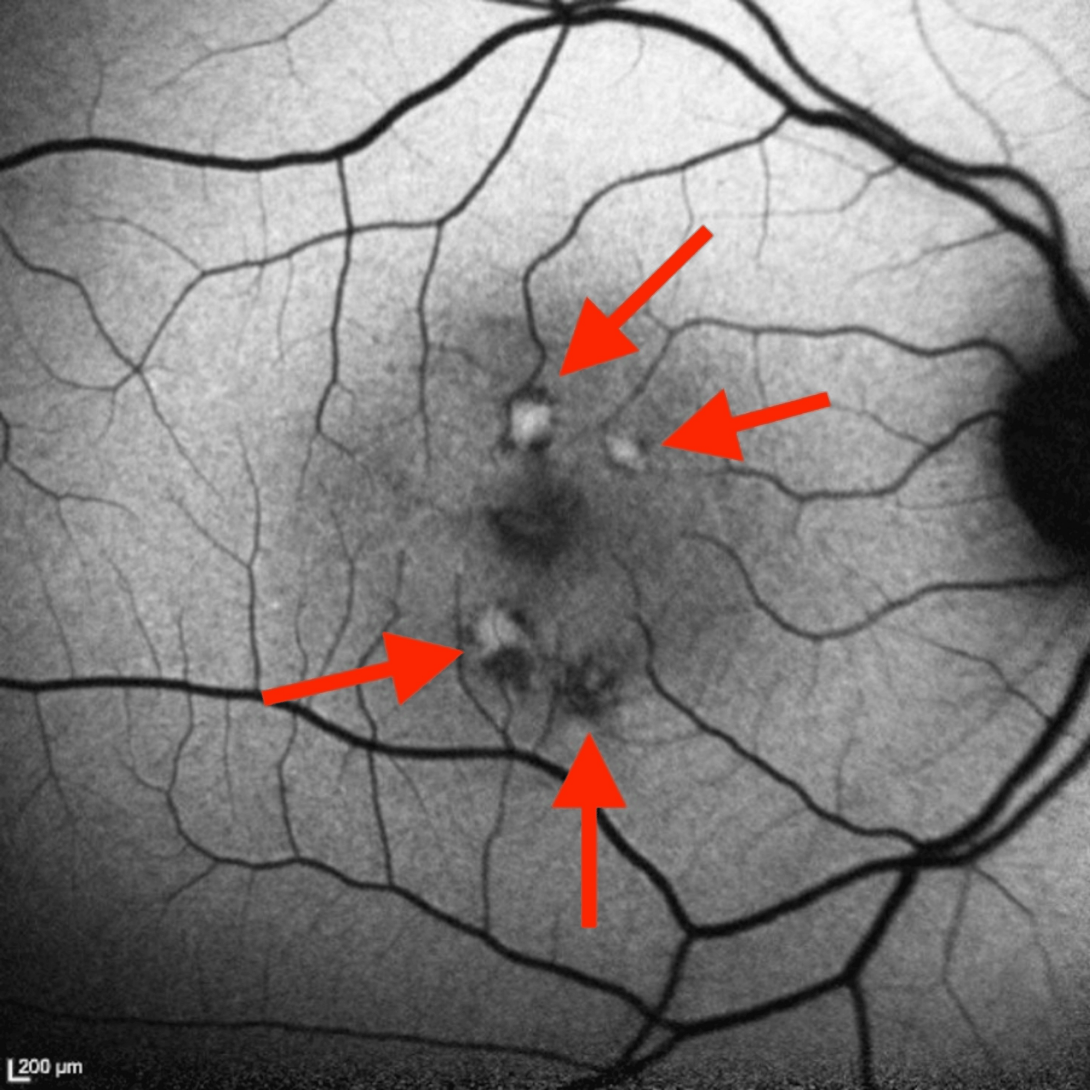
Fundus Autofluorescence of the Right Eye From the Six-Month Ophthalmology Follow-Up

**Figure 8:**
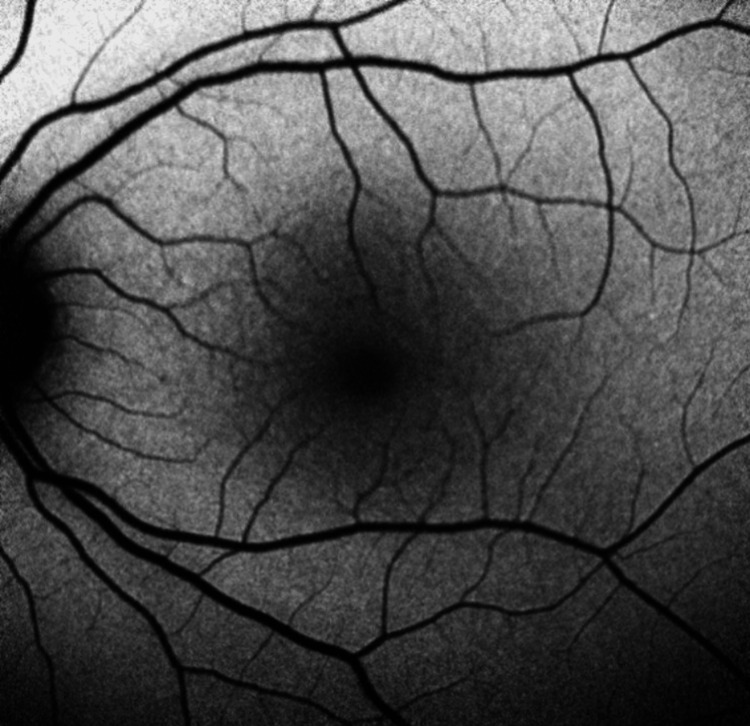
Fundus Autofluorescence of the Left Eye From the Six-Month Ophthalmology Follow-Up

The patient was advised to do Amsler Grid monitoring regularly, start age-related eye disease 2 (AREDS2) supplements, follow a Mediterranean diet, and wear sunglasses outdoors. He was compliant and continued to have regular follow-up appointments with his PCP and ophthalmologist. At his six-month follow-up with the ophthalmologist, diagnostic and digital retinal exams (Figures 7-8) revealed no further progression of AMD bilaterally, and he was counseled to continue following the treatment plan.

## Discussion

Given the various risk factors associated with AMD, it is uncommon for a patient in their 40s who does not smoke, rarely consumes alcohol, and has no family history of AMD to be diagnosed with the condition. Considering that AMD is a leading cause of vision loss, healthcare professionals must emphasize the importance of baseline eye exams for patients beginning at the age of 40 [3,10].

This case highlights the current treatment options available for intermediate AMD, including AREDS2 supplements and dietary modifications. The AREDS2 supplement consists of a daily dose of 500 mg of vitamin C, 400 international units (IU) of vitamin E, 80 mg of zinc oxide, 2 mg of cupric oxide, 10 mg of lutein, 2 mg of zeaxanthin, and 1 g of omega-3 fatty acids [5]. A clinical trial of high-dose supplementation with these ingredients revealed significantly reduced odds of developing advanced AMD in the higher-risk group in comparison with the placebo group used in the study [3]. The patient adhered to the treatment plan, and it was encouraging to see that at the six-month follow-up, there was no further progression of AMD. Early detection and proactive management can delay the progression of AMD, helping prevent further vision loss and preserve the quality of life in a young patient.

## Conclusions

This case demonstrates the critical role of regular primary care visits in establishing continuity of care and facilitating routine laboratory screening. Despite the patient’s perception of good health, his labs revealed significant findings, including elevated LDL, reduced HDL, and a prediabetic HbA1c. Primary care providers are well-positioned to address seemingly minor concerns, perform appropriate evaluations, and initiate timely referrals to specialists when indicated. Although this patient had an overall insignificant past medical history and was nearsighted, his retinal images at the optometry visit were concerning and prompted him to speak to his PCP and visit an ophthalmologist. This case also serves as a reminder that clinical presentations normally seen in the geriatric populations should not be excluded in younger adults presenting with mild symptoms outside the expected demographic for such diseases.
